# A Review of Cardiovascular Complications among Pregnant Patients with COVID-19

**DOI:** 10.31083/j.rcm2311383

**Published:** 2022-11-16

**Authors:** Alix J. Pruzansky, Justin J. Slade, Megan Stephenson, Seema Pursnani

**Affiliations:** ^1^Department of Cardiology, Kaiser Permanente, Santa Clara, CA 95051, USA; ^2^Department of Cardiology, Kaiser Permanente, San Francisco, CA 94115, USA; ^3^Department of Maternal Fetal Medicine, Kaiser Permanente, Santa Clara, CA 95051, USA

**Keywords:** COVID-19, pregnancy, cardiovascular complications

## Abstract

Cardiovascular complications of severe acute respiratory syndrome corona virus 2 (SARS-CoV-2) infection are well-described in the general population but remain limited among pregnant patients. This review summarizes data from case reports, case series, and observational studies of cardiovascular manifestations of corona virus disease 2019 (COVID-19) in pregnant patients and provides recommendations to the cardiovascular clinician regarding management considerations in this vulnerable population. Pregna is an immunocompromised state in which cardiovascular demands are increased. Cardiovascular complications of COVID-19 that have been described in pregnancy include myocardial injury, cardiomyopathy, thromboembolism, pre-eclampsia and arrhythmia. Physiologic and cardiovascular changes in pregnancy predispose pregnant patients with COVID-19 to more severe illness than the general population. Black or Hispanic race, obesity, diabetes, hypertension and lung disease are risk factors for more severe infection, maternal death and adverse perinatal outcomes. Pregnant patients with severe COVID-19 disease compared with non-pregnant age-matched women with COVID infection are more likely to be admitted to the intensive care unit (ICU), receive mechanical ventilation and require advanced mechanical circulatory support. Cardiovascular complications of COVID-19 in pregnant patients requires further attention, particularly given the anticipated increase in birth volume and ongoing nature of COVID-19 pandemic with novel variants. Clinicians should have a lower threshold for cardiac testing and multidisciplinary management in pregnant women with severe COVID-19 disease. Given the persistence of COVID-19 within our communities, diagnostic laboratory and imaging testing for high-risk pregnant patients hospitalized with COVID-19 infection should be routine. We strongly urge the implementation of a cardio-obstetric multidisciplinary team in individually managing these high-risk patients in an effort to improve maternal and fetal outcomes.

## 1. Introduction 

The coronavirus disease of 2019 (COVID-19), caused by severe acute respiratory syndrome coronavirus 2 (SARS-CoV-2), was declared a pandemic by the World Health Organization in March 2020 [1]. As of May 2022, there have been an estimated 524 million confirmed cases of COVID-19 worldwide with over six million deaths [2]. Sparse data exists regarding the effects of COVID-19 on maternal health during pregnancy. Pregnancy is associated with physiologic alterations in immune regulation that increase susceptibility to infectious respiratory organisms including influenza and other SARS infections [3]. Symptomatic pregnant patients, compared to non-pregnant patients with COVID-19, are at increased risk of more severe illness due to COVID-19 including preeclampsia, preterm delivery, and maternal mortality [4]. Compared with non-pregnant age-matched women with COVID-19, parturients with severe COVID-19 in pregnancy are more likely to be admitted to the intensive care unit, require mechanical ventilation, or necessitate advanced respiratory and hemodynamic support, including extracorporeal membrane oxygenation (ECMO) [5, 6, 7, 8, 9]. Pregnant and postpartum patients who are Black or Hispanic, of advanced maternal age, or those with comorbidities including obesity, diabetes, hypertension and lung disease may be at even higher risk for severe COVID-19 infection, maternal death, and adverse perinatal outcomes including increased cesarean delivery and hypertensive disorders of pregnancy (HDP) [10, 11, 12, 13].

While respiratory infection and resulting systemic illness are the most common clinical manifestations of COVID-19, cardiovascular complications have been increasingly recognized, which include myocardial injury, cardiomyopathy, thromboembolism (TE), preeclampsia and other HDP, and arrhythmias [14, 15, 16, 17, 18]. Of note, several case series and cohort studies evaluating the cardiovascular outcomes of COVID-19 in pregnancy were performed in the early months of the pandemic (March to June 2020) when morbidity and mortality were higher [19, 20, 21, 22, 23]. However, given the anticipated increase in birth volume and the ongoing nature of the COVID-19 pandemic with novel variants, understanding cardiovascular complications of severe COVID-19 disease remains relevant to the cardiovascular clinician. In this review, we seek to consolidate available data reported to date of cardiovascular manifestations of COVID-19 in pregnant patients, provide recommendations in clinical management, and anticipate future investigative needs in this vulnerable population. Papers for this review were selected through online search in databases of PubMed using appropriate search terms as well as reference lists of retrieved papers, all in the English language.

## 2. Physiologic Cardiovascular Changes in Pregnancy

Understanding relevant physiologic cardiovascular changes during pregnancy allows for an understanding of abnormalities in the setting of systemic illness. Cardiac output increases throughout pregnancy, with the sharpest rise in the first and second trimester, with up to a 45% increase in a singleton pregnancy and 60% increase in a twin pregnancy [24]. This increased cardiac output is mediated by both an increase in stroke volume and heart rate [25]. Heart rate increases progressively during normal gestation, reaching a maximum in the third trimester, with an approximate 20% to 25% increase over preconception baseline levels [25]. There is a decrease in arterial systolic and diastolic pressures during pregnancy with the lowest values in the second trimester, dropping 5–10 mm Hg below baseline, followed by return to preconception values in the early postpartum period [25]. Echocardiography studies have demonstrated temporary cardiac remodeling with left and right ventricular wall thickness and left ventricular mass increasing throughout pregnancy [26]. Myocardial contractility and left and right ventricular ejection fractions remain largely unchanged during pregnancy [27, 28]. During a normotensive pregnancy, troponin I (cTnI) and creatine kinase-MB (CK-MB) biomarkers are unchanged and unaffected by labor, anesthesia, or cesarean section [29]. B-type natriuretic peptide levels (BNP) remain within normal limits in a normal pregnancy but will be abnormal in congestive heart failure. D-dimer levels rise steadily during pregnancy and can rise to 96–100% beyond the non-pregnancy threshold by the third trimester [30].

## 3. COVID-19 and Cardiac Involvement in Pregnancy

### 3.1 Myocardial Injury

Myocardial injury, evidenced by elevated cardiac biomarkers, is among the most widely reported cardiac manifestation of COVID-19 [31, 32], with an estimated 33% of critically ill nonpregnant patients showing significantly elevated cardiac biomarkers [33, 34]. Myocardial injury reported among patients with COVID-19 can be due to myocarditis [35], or atherosclerotic plaque rupture leading to coronary thrombosis and acute myocardial infarction [36]. Troponin I elevations in hospitalized patients with COVID-19 are common and the degree of elevation can be a predictor of mortality [37]. Similarly, a normal troponin-I level in the first 24 hours of admission has a high negative predictive value for all-cause in-hospital mortality [38]. Mechanisms for developing myocardial injury in the setting of COVID-19 infection are not fully understood, but one hypothesis is virus-mediated lysis of cardiomyocytes, which has also been observed in other viral infections, or as a consequence of SARS-CoV-2 binding angiotensin converting enzyme 2 (ACE2) receptors in the heart [39]. Other proposed mechanisms include microvascular dysfunction, multisystem immune-mediated or stress-mediated dysfunction with elevated inflammatory markers, cytokine storm, and hypoxia-induced cardiac myocyte apoptosis [40, 41]. Myocardial injury due to COVID-19 may also be due to supply-demand mismatch (type 2 myocardial infarction), particularly where there is not a concomitant cardiomyopathy.

Myocardial injury among pregnant patients with COVID-19 has been described in a limited number of retrospective studies and case reports. Mercedes *et al*. [19] evaluated 154 pregnant patients with confirmed COVID-19 admitted to a single tertiary care hospital in the Dominican Republic between March and June 2020 to evaluate maternal and fetal clinical outcomes. Of this cohort, 34 patients (22%) had severe disease requiring intensive care unit (ICU) level care and 15 (9.7%) developed myocardial injury with left ventricular systolic dysfunction. Of these patients, all had elevated cardiac biomarkers, with a median troponin 34.6 ng/mL and mean left ventricular ejection fraction (LVEF) 37.7% ± 6.4% with a predominant pattern of diffuse global hypokinesis. All patients were delivered by cesarean section, and 60% of births were preterm (mean gestational age at delivery was 34.2 weeks ± 4 weeks). Two patients developed fatal ventricular arrythmias (ventricular tachycardia and torsade de pointes), leading also to one fetal demise. In a cohort of 31 pregnant patients hospitalized for severe COVID-19 at 7 hospitals in a large healthcare system in New York, 20 patients (65%) had cardiac biomarker testing, which was elevated in four (22%) [20]. Only four patients had transthoracic echocardiograms (TTE) performed, and all were reported normal. No patients had preexisting cardiovascular disease or hypertension.

Myocarditis and myopericarditis have been reported both from COVID-19 infection [42, 43], after COVID-19 recovery [44], and in association with COVID-19 mRNA vaccines [45, 46, 47, 48], but there are no existing reports in pregnancy (as of May 2022). However, given the variability in diagnostic criteria for myocarditis (endomyocardial biopsy, advanced cardiac imaging), this certainly could be underdiagnosed and underreported and has yet to be reported in pregnancy registries.

**Fig. 1.**
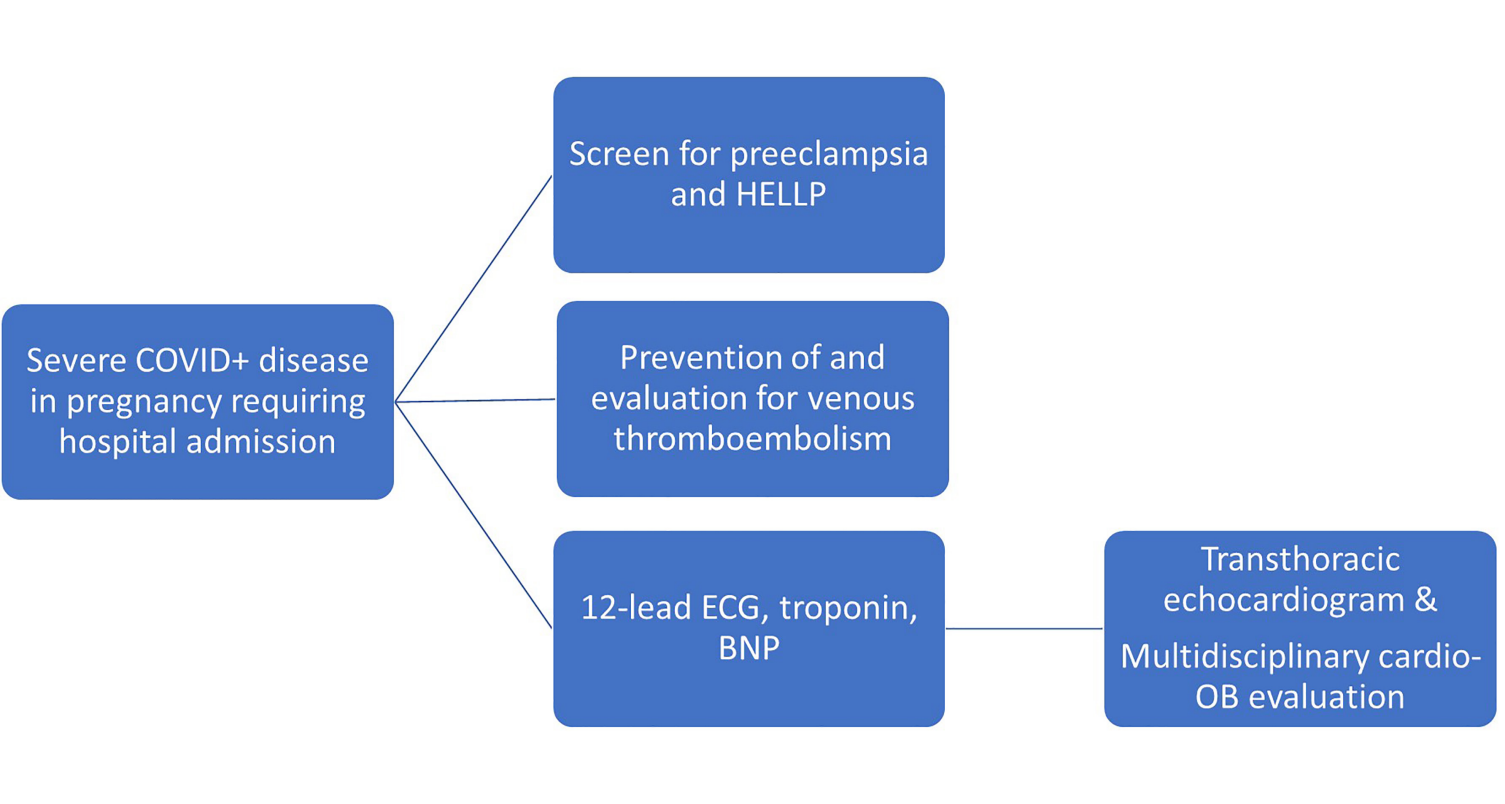
**A framework for addressing cardiovascular complications associated with COVID**-**19 among hospitalized pregnant patients**.

Because COVID-19 during pregnancy is associated with greater morbidity and mortality, we would advise a low threshold to evaluate and trend cardiac biomarkers to guide additional workup among hospitalized pregnant patients with COVID-19 (Fig. 1).

### 3.2 Cardiomyopathy

The presenting symptoms of COVID-19, including cough and dyspnea, can mimic those of acute congestive heart failure (CHF). In one series of 113 deceased patients, CHF occurred in one quarter of non-pregnant COVID-19 patients [49]. Cardiomyopathies among pregnant COVID-19 patients may be due to a peripartum [50, 51], sepsis or stress mediated (Takotsubo) cardiomyopathy [52]. Peripartum cardiomyopathy (PPCM) is a diagnosis of exclusion and differentiating between PPCM and COVID-19 mediated cardiomyopathy may prove to be clinically challenging. A proposed mechanism of COVID-19 mediated cardiomyopathy is cytokine release syndrome (CRS), an excessive and dysregulated systemic inflammatory response from the viral insult, which has previously been seen during Middle East Respiratory Syndrome (MERS) and SARS epidemics [53, 54, 55]. Ye *et al*. [56] studied the pathogenesis of CRS in COVID-19 and report high levels of proinflammatory cytokines, tumor necrosis factor and chemokines that attract inflammatory cells resulting in massive infiltration of parenchymal, including cardiac, tissue throughout the body. Studies show that levels of the inflammatory marker IL-6 correlate positively with the severity of disease [57].

The development of heart failure with reduced ejection (HFrEF) in the peripartum woman with COVID-19 may be multifactorial in etiology [58]. Juusela *et al*. [21] presented a limited case series where two of the first seven pregnant patients at a single tertiary care institution confirmed with COVID-19 in March 2020 developed respiratory symptoms and mild to moderately reduced left ventricular ejection fraction of 40–45% with global hypokinesis, but no initial evidence of myocardial injury. One case was complicated by a cardiac arrest requiring cardiopulmonary resuscitation and another by transient but stable supraventricular tachycardia. Both patients had risk factors for cardiac complications including Black and Hispanic race, obesity, and one patient was of advanced maternal age. Both developed acute respiratory complications and were delivered by emergent cesarean section at 33 and 39 weeks respectively. The study was limited by small sample size and follow-up limited to the hospital encounter. In a second study, among a cohort of 64 hospitalized pregnant patients with COVID-19 across twelve United States institutions, 17% had known cardiac disease and their mean BMI was 34 kg/$m^{2}$; in this series, there were no reported cases of cardiomyopathy or maternal death, but one patient suffered a successfully resuscitated cardiac arrest [22].

Stress induced (Takotsubo) cardiomyopathy has been widely reported among nonpregnant COVID-19 patients [59, 60], but only in a single case report of early pregnancy in a patient with COVID-19. Bhattacharyya *et al*. [52] reported a case of a 32-year old COVID-19 positive primigravida at 38 weeks gestation with a history of gestational hypertension who presented with three days of palpitations. Cardiac biomarkers were normal and a TTE demonstrated hypokinetic apical left ventricular wall segments with reduced ejection fraction and hypercontractile basal segments with prominent apical ballooning typical for a Takotsubo cardiomyopathy. Conservative management, including initiation of a beta blocker, led to complete clinical improvement and normalization of left ventricular systolic function. The patient delivered at term and without additional complications.

We suggest routine evaluation of cardiac troponin and BNP levels in hospitalized pregnant patients with severe COVID-19 infection given the higher risk for cardiovascular complications and potential for hemodynamic deterioration. Positive values can guide the need for obtaining a TTE and subsequent additional cardiac workup.

### 3.3 Thromboembolic Events

Thromboembolism (TE) includes both venous and arterial clotting disorders such as acute pulmonary embolism, ischemic stroke, deep vein thrombosis or myocardial infarction (MI). Pregnancy and the postpartum period confer a hypercoagulable state, with a 4–6 fold increased risk of TE in the third trimester [61]. This risk is further increased if a pregnant woman is overweight or obese, older than 35-years age, or hospitalized for more than three days [62]. The frequency of TE among nonpregnant adults admitted to the intensive care unit with COVID-19 was 25%–31% [63, 64] and associated with higher mortality [65]. Prevalence of TE in the pregnant population is limited to a single cohort study and several case reports [22, 66, 67, 68]. Due to the combined hypercoagulable states of pregnancy and that conferred by COVID-19 infection, pregnant patients have an increased risk of fatal TE events [69]. Mechanisms of hypercoagulability in pregnancy include progesterone mediated increase in venous capacitance and mechanical compression by a gravid uterus leading to reduced venous outflow [70]. In the setting of COVID-19 infection, endothelial cell dysfunction from angiotensin-converting enzyme 2 proteins, hypercoagulability from an overwhelming inflammatory state, altered blood flow from elevated fibrinogen and stasis in a hospitalized patient can further contribute to TE. During normal pregnancy, D-dimer and fibrinogen levels increase progressively and peak in the third trimester [71]. The hypercoagulable state inherent to pregnancy makes interpretation of coagulation tests of the pregnant COVID-19 patient difficult to interpret. Systemic inflammation and coagulopathy in COVID-19 can theoretically increase the risk of atherosclerotic plaque rupture with acute MI [17], although no studies to date have reported MI among pregnant patients with COVID-19.

Jering *et al*. [22] examined 406,446 patients from a large national cohort of US patients hospitalized for childbirth over an eight-month period and found that 6380 (1.6%) were COVID-19 positive. Of the cohort with COVID-19 who gave birth, 212 (3.3%) required intensive care, of whom 86 (1.3%) required mechanical ventilation and 9 (0.1%) died. Rates of MI and TE were higher in the patients with, versus without, COVID-19 (MI: 0.1% *vs* 0.004%; VTE: 0.2% *vs* 0.1%; *p *< 0.001). Additional cases of presumed COVID-19 induced coagulopathy in pregnancy, including pulmonary embolism, ovarian vein thrombosis, and other adverse thrombotic complications, have been reported [66, 67, 68]. Ongoing data collection in an international registry aims to guide the management of COVID-19 and associated coagulopathy in pregnancy [69]. To date, there are no dedicated studies evaluating VTE outcomes among pregnant patients who have received COVID-19 vaccines although this has been increasingly studied in the general population. A large US observational cohort of 792,010 patients who received at least one authorized COVID-19 vaccine (BNT162b2—Pfizer-BioNTech, mRNA-1273—Moderna, and Ad.26.COV2.S—Janssen/Johnson &Johnson) had no significant elevation in VTE risk post vaccination [68].

Similar to the nonpregnant population, we suggest consideration of weight adjusted TE prophylaxis with low molecular weight heparin in all symptomatic pregnant and post-partum patients requiring hospitalization for COVID-19 infection, in the absence of maternal or fetal contraindications to anticoagulation use.

### 3.4 Preeclampsia

Preeclampsia is defined as new onset hypertension and proteinuria, or new onset hypertension and significant end-organ dysfunction with or without proteinuria after 20 weeks of gestation in a previously normotensive woman [72]. COVID-19 has been shown to increase the risk of pre-eclampsia and, in turn, adverse pregnancy outcomes [73, 74]. COVID-19 and pre-eclampsia share many common risk factors such as obesity and pre-existing systemic hypertension. Preeclampsia is a syndrome of systemic maternal endothelial dysfunction driven by excess of circulation antiangiogenic proteins in the setting of a susceptible mother [75]. Evidence suggests that SARS-CoV-2 also causes endothelial dysfunction and promotes a procoaguable state similar to that seen in preeclampsia and the use of the term “preeclampsia-like syndrome” describes the clinical ambiguity in distinguishing the two syndromes [76].

In a systematic review and meta-analysis of 42 observational studies involving 438,548 pregnant women, COVID-19 infection in pregnancy was associated with higher rates of preeclampsia (odd ratio [OR] 1.33, 95% confidence interval [CI]: 1.03, 1.73) compared to pregnant patients without COVID-19 infection [77]. Those with severe disease had stronger associations with preeclampsia compared to those with mild illness (OR 4.16, 95% CI: 1.55, 11.15). Several case reports also describe severe preeclampsia and HELLP (hemolysis, elevated liver enzymes and low platelets) syndrome in pregnant patients with COVID-19 infection [78, 79, 80]. In the INTERCOVID study, a longitudinal, prospective, unmatched observational study, Papageorghiou *et al*. [81] found that COVID-19 during pregnancy was strongly associated with preeclampsia independent of any risk factors and preexisting conditions (risk ratio, 1.77; 95% CI: 1.25, 2.52). Emerging data suggests that pregnant patients with COVID-19 who develop this preeclampsia like syndrome may be distinguished from traditional preeclampsia by soluble fms-like tyrosine kinase-1 (sFlt-1) [82], though this association requires further review and validation.

Pregnant individuals who have preexisting hypertension, obesity, or diabetes are at an increased risk for preeclampsia and should also be considered high risk for severe COVID-19 infection. Many of these patients with additional risk factors for preeclampsia will be on daily low dose aspirin (81 mg) as part of the US Preventative Task Force (USPSTF) recommendations for prevention of preeclampsia in high-risk populations [83].

At this time, we recommend that intensive blood pressure monitoring in those pregnant patients diagnosed with COVID-19 is essential to the prompt recognition and management of preeclampsia.

### 3.5 Cardiac Arrhythmia and Cardiac Arrest

Arrhythmia is a common cardiovascular complication among nonpregnant patients with severe COVID-19. Incidence and type of arrhythmia vary among studies. Among 138 hospitalized patients with severe COVID-19 at Zhongnan Hospital in Wuhan, China, up to 44% of intensive care unit patients developed an arrhythmia, although subtypes of arrhythmia were not detailed [84]. Malignant ventricular arrhythmias, including ventricular tachycardia and ventricular fibrillation have been reported in up to 5.9% of nonpregnant patients hospitalized with severe COVID-19 [40]. Among 700 nonpregnant adults admitted due to COVID-19 infection from a single US institution, Bhatla *et al*. [85] reported a 7.5% overall incidence of arrhythmias, of which 43% occurred among patients admitted to the ICU. Arrhythmias included: 9 with ventricular arrhythmia leading to cardiac arrest, 25 with atrial fibrillation, 9 with bradyarrhythmias, and 10 with non-sustained ventricular tachycardias [85]. There are many features of COVID-19 and critical illness that predispose patients to proarrhythmic states. Systemic infection and inflammation from the overproduction of proinflammatory cytokines including IL-6, critical illness, and profound hypoxia are all potential mechanisms leading to ventricular arrhythmias and sudden cardiac death. Arrythmias in COVID-19 may also be secondary to medication side effects including polypharmacy, impaired drug clearance due to critical illness, and QT prolongation [86].

Broadly, arrhythmias are common in pregnancy, often due to benign premature atrial or ventricular contractions or paroxysmal supraventricular tachycardia. Less common are atrial fibrillation or ventricular tachycardia. While various arrhythmias among pregnant COVID-19 patients have been reported as sequelae or incidental finding in case reports [19, 21], there are no dedicated studies that have assessed the incidence of arrhythmia or cardiac arrest in this particular sub-population.

We recommend, at a minimum, a 12 lead electrocardiogram in all hospitalized pregnant COVID-19 patients. In those with known cardiac disease, electrolyte abnormalities or need for drugs that may prolong the QT interval, serial monitoring is appropriate. Consideration for the need for additional therapies, such asexample anticoagulation if atrial fibrillation occurs, or a wearable lifevest, if ventricular arrhythmias are present, is crucial. Patients with sustained torsades de pointes (TdP) or who become hemodynamically unstable, should be treated according to standard resuscitation algorithms including cardioversion and defibrillation. Guideline based interventions for unstable arrhythmias should not be held or delayed in the setting of pregnancy with COVID-19 infection, and pregnant patients should be treated expeditiously with appropriate use of personal protective equipment.

## 4. Study Limitations

Certain limitations of this review and the data presented should be noted. Overall, there is a striking paucity of data available in this population. The majority of studies evaluating the cardiovascular outcomes of COVID-19 in pregnancy, particularly those describing myocardial injury and cardiomyopathy, were reported in the early months of the pandemic (March to June 2020) when morbidity and mortality were higher and the population was largely unvaccinated [19, 20, 21, 22, 23]. There are no data describing whether outcomes have improved among pregnant COVID-19 patients with severe disease since the beginning of the pandemic. A report by the Centers for Disease Control and Prevention published in January 2022 showed that among patients with COVID-19 infection from the SARS-CoV-2 B.1.1.529 (Omicron) variant that became predominant in the United States in December 2021 with the highest reported numbers of cases and hospitalizations, disease severity indicators, such as length of stay, ICU admission, and death, were lower than during pervious pandemic peaks [87]. Further, SARS-CoV-2 has undergone numerous mutations and different variants have become predominant at various times and in differing geographic places. It is unclear if certain strains carry greater risk for cardiovascular complications, as genotype data was not routinely collected in the studies presented.

Lastly, in most reported cases of cardiovascular complications in pregnancy, patients with COVID-19 simultaneously presented with severe or critical illness, and the cardiovascular insults may be due to the multisystem inflammatory syndrome, SARS-CoV-2 virus infection itself, the uncovering of previously undiagnosed heart conditions, and/or a multifactorial process. The rapidly evolving landscape of vaccinations, viral mutations, and lack of large studies among pregnant women with severe COVID-19 infection highlights the necessity for continued studies in this area.

## 5. Conclusions and Future Directions

In summary, myocardial injury, cardiomyopathy, thromboembolic events, preeclampsia and arrhythmias are among the most reported cardiovascular complications of COVID-19 in the pregnant population. Pregnancy induces physiologic changes that have a significant impact on the immune system, respiratory system, coagulation cascade, and cardiovascular function, placing this population at increased risk of severe COVID-19 infection. Due to the ongoing presence of new COVID-19 variants and the lack of data in this high-risk population, there is a pressing need for the systematic study of maternal and fetal outcomes, including cardiovascular complications, of COVID-19 in pregnant compared to non-pregnant populations. Several registries have been created [88], including the Pregnancy Coronavirus Outcomes Registry (PRIORITY) [89], and INTERCOVID: A prospective cohort study of the effects of COVID-19 in pregnancy and the neonatal period [90] Registry creation is key to generating more robust data to guide practice.

Although data is limited primarily to case reports, series and retrospective cohort studies, available literature suggests that pregnancy is a risk factor for higher rates of severe COVID-19 disease and its complications. In light of the dynamic and ongoing nature of the pandemic, our understanding of COVID-19 infection in the pregnant patient and its impact on maternal and fetal well-being is evolving. During the early periods of the pandemic, many institutions took a minimalist approach to evaluation and testing of patients to preserve personnel, personal protection equipment, and for infection control. Given the persistence of COVID-19 within our communities, diagnostic laboratory and imaging testing for pregnant patients hospitalized with severe COVID-19 infection should be considered routine. Specifically, medical teams should have a low threshold to obtain a 12 lead electrocardiogram and cardiac biomarkers (CTn, BNP) on all pregnant patients with severe COVID-19 infection to help guide the need for further imaging by TTE. Consultation with a multi-disciplinary cardio-obstetrics team can be obtained, to guide subsequent clinical decisions and in an effort to improve maternal and fetal outcomes.

## References

[1] Cucinotta D, Vanelli M (2020). WHO Declares COVID-19 a Pandemic. *Acta Bio-Medica: Atenei Parmensis*.

[2] World Health Organization WHO Coronavirus (COVID-19) Dashboard. https://covid19.who.int/.

[3] Wong SF, Chow KM, Leung TN, Ng WF, Ng TK, Shek CC (2004). Pregnancy and perinatal outcomes of women with severe acute respiratory syndrome. *American Journal of Obstetrics and Gynecology*.

[4] Villar J, Ariff S, Gunier RB, Thiruvengadam R, Rauch S, Kholin A (2021). Maternal and Neonatal Morbidity and Mortality among Pregnant Women with and without COVID-19 Infection: The INTERCOVID Multinational Cohort Study. *JAMA Pediatrics*.

[5] Ellington S, Strid P, Tong VT, Woodworth K, Galang RR, Zambrano LD (2020). Characteristics of Women of Reproductive Age with Laboratory-Confirmed SARS-CoV-2 Infection by Pregnancy Status—United States, January 22–June 7, 2020. *Obstetrical & Gynecological Survey*.

[6] Zambrano LD, Ellington S, Strid P, Galang RR, Oduyebo T, Tong VT (2020). Update: Characteristics of Symptomatic Women of Reproductive Age with Laboratory-Confirmed SARS-CoV-2 Infection by Pregnancy Status - United States, January 22-October 3, 2020. *Morbidity and Mortality Weekly Report*.

[7] Khan DSA, Pirzada AN, Ali A, Salam RA, Das JK, Lassi ZS (2021). The Differences in Clinical Presentation, Management, and Prognosis of Laboratory-Confirmed COVID-19 between Pregnant and Non-Pregnant Women: A Systematic Review and Meta-Analysis. *International Journal of Environmental Research and Public Health*.

[8] Fisher SA, Goldstein JA, Mithal LB, Isaia AL, Shanes ED, Otero S (2021). Laboratory analysis of symptomatic and asymptomatic pregnant patients with SARS-CoV-2 infection. *American Journal of Obstetrics & Gynecology MFM*.

[9] Ko JY, DeSisto CL, Simeone RM, Ellington S, Galang RR, Oduyebo T (2021). Adverse Pregnancy Outcomes, Maternal Complications, and Severe Illness among us Delivery Hospitalizations with and without a Coronavirus Disease 2019 (COVID-19) Diagnosis. *Clinical Infectious Diseases*.

[10] Onwuzurike C, Diouf K, Meadows AR, Nour NM (2020). Racial and ethnic disparities in severity of COVID‐19 disease in pregnancy in the United States. *International Journal of Gynecology & Obstetrics*.

[11] Metz TD, Clifton RG, Hughes BL, Sandoval G, Saade GR, Grobman WA (2021). Disease Severity and Perinatal Outcomes of Pregnant Patients With Coronavirus Disease 2019 (COVID-19). *Obstetrics & Gynecology*.

[12] Allotey J, Stallings E, Bonet M, Yap M, Chatterjee S, Kew T (2020). Clinical manifestations, risk factors, and maternal and perinatal outcomes of coronavirus disease 2019 in pregnancy: living systematic review and meta-analysis. *British Medical Journal*.

[13] Lassi ZS, Ana A, Das JK, Salam RA, Padhani ZA, Irfan O (2021). A systematic review and meta-analysis of data on pregnant women with confirmed COVID-19: Clinical presentation, and pregnancy and perinatal outcomes based on COVID-19 severity. *Journal of Global Health*.

[14] Alizadehsani R, Eskandarian R, Behjati M, Zahmatkesh M, Roshanzamir M, Izadi NH (2022). Factors associated with mortality in hospitalized cardiovascular disease patients infected with COVID‐19. *Immunity, Inflammation and Disease*.

[15] Linschoten M, Peters S, van Smeden M, Jewbali LS, Schaap J, Siebelink H (2020). Cardiac complications in patients hospitalised with COVID-19. *European Heart Journal. Acute Cardiovascular Care*.

[16] Golemi Minga I, Golemi L, Tafur A, Pursnani A (2020). The Novel Coronavirus Disease (COVID-19) and its Impact on Cardiovascular Disease. *Cardiology in Review*.

[17] Long B, Brady WJ, Koyfman A, Gottlieb M (2020). Cardiovascular complications in COVID-19. *The American Journal of Emergency Medicine*.

[18] Farshidfar F, Koleini N, Ardehali H (2021). Cardiovascular complications of COVID-19. *JCI Insight*.

[19] Mercedes BR, Serwat A, Naffaa L, Ramirez N, Khalid F, Steward SB (2021). New-onset myocardial injury in pregnant patients with coronavirus disease 2019: a case series of 15 patients. *American Journal of Obstetrics and Gynecology*.

[20] Pachtman Shetty SL, Meirowitz N, Blitz MJ, Gadomski T, Weinberg CR (2021). Myocardial injury associated with coronavirus disease 2019 in pregnancy. *American Journal of Obstetrics and Gynecology*.

[21] Juusela A, Nazir M, Gimovsky M (2020). Two cases of coronavirus 2019-related cardiomyopathy in pregnancy. *American Journal of Obstetrics & Gynecology MFM*.

[22] Jering KS, Claggett BL, Cunningham JW, Rosenthal N, Vardeny O, Greene MF (2021). Clinical Characteristics and Outcomes of Hospitalized Women Giving Birth with and without COVID-19. *JAMA Internal Medicine*.

[23] Pierce-Williams RAM, Burd J, Felder L, Khoury R, Bernstein PS, Avila K (2020). Clinical course of severe and critical coronavirus disease 2019 in hospitalized pregnancies: a United States cohort study. *American Journal of Obstetrics & Gynecology MFM*.

[24] Hunter S, Robson SC (1992). Adaptation of the maternal heart in pregnancy. *Heart*.

[25] Sanghavi M, Rutherford JD (2014). Cardiovascular Physiology of Pregnancy. *Circulation*.

[26] Poppas A, Shroff SG, Korcarz CE, Hibbard JU, Berger DS, Lindheimer MD (1997). Serial assessment of the cardiovascular system in normal pregnancy: role of arterial compliance and pulsatile arterial load. *Circulation*.

[27] Umar S, Nadadur R, Iorga A, Amjedi M, Matori H, Eghbali M (2012). Cardiac structural and hemodynamic changes associated with physiological heart hypertrophy of pregnancy are reversed postpartum. *Journal of Applied Physiology*.

[28] Ducas RA, Elliott JE, Melnyk SF, Premecz S, daSilva M, Cleverley K (2014). Cardiovascular magnetic resonance in pregnancy: Insights from the cardiac hemodynamic imaging and remodeling in pregnancy (CHIRP) study. *Journal of Cardiovascular Magnetic Resonance*.

[29] Koscica KL, Bebbington M, Bernstein PS (2004). Are maternal serum troponin I levels affected by vaginal or cesarean delivery. *American Journal of Perinatology*.

[30] Morton A (2021). Physiological Changes and Cardiovascular Investigations in Pregnancy. *Heart, Lung and Circulation*.

[31] Shi S, Qin M, Shen B, Cai Y, Liu T, Yang F (2020). Association of Cardiac Injury with Mortality in Hospitalized Patients with COVID-19 in Wuhan, China. *JAMA Cardiology*.

[32] Mishra AK, Sahu KK, George AA, Lal A (2020). A review of cardiac manifestations and predictors of outcome in patients with COVID-19. *Heart & Lung*.

[33] Guo T, Fan Y, Chen M, Wu X, Zhang L, He T (2020). Cardiovascular Implications of Fatal Outcomes of Patients with Coronavirus Disease 2019 (COVID-19). *JAMA Cardiology*.

[34] Arentz M, Yim E, Klaff L, Lokhandwala S, Riedo FX, Chong M (2020). Characteristics and Outcomes of 21 Critically Ill Patients with COVID-19 in Washington State. *Journal of the American Medical Association*.

[35] Cheng R, Leedy D (2020). COVID-19 and acute myocardial injury: the heart of the matter or an innocent bystander. *Heart*.

[36] Bansal M (2020). Cardiovascular disease and COVID-19. *Diabetes & Metabolic Syndrome: Clinical Research & Reviews*.

[37] Zhou F, Yu T, Du R, Fan G, Liu Y, Liu Z (2020). Clinical course and risk factors for mortality of adult inpatients with COVID-19 in Wuhan, China: a retrospective cohort study. *The Lancet*.

[38] AL Abbasi B, Torres P, Ramos-Tuarez F, Dewaswala N, Abdallah A, Chen K (2020). Cardiac Troponin-I and COVID-19: a Prognostic Tool for in-Hospital Mortality. *Cardiology Research*.

[39] Page EM, Ariëns RAS (2021). Mechanisms of thrombosis and cardiovascular complications in COVID-19. *Thrombosis Research*.

[40] Driggin E, Madhavan MV, Bikdeli B, Chuich T, Laracy J, Biondi-Zoccai G (2020). Cardiovascular Considerations for Patients, Health Care Workers, and Health Systems During the COVID-19 Pandemic. *Journal of the American College of Cardiology*.

[41] Clerkin KJ, Fried JA, Raikhelkar J, Sayer G, Griffin JM, Masoumi A (2020). Coronavirus disease 2019 (COVID-19) and cardiovascular disease. *Circulation*.

[42] Doyen D, Moceri P, Ducreux D, Dellamonica J (2020). Myocarditis in a patient with COVID-19: a cause of raised troponin and ECG changes. *The Lancet*.

[43] Paul JF, Charles P, Richaud C, Caussin C, Diakov C (2020). Myocarditis revealing COVID-19 infection in a young patient. *European Heart Journal: Cardiovascular Imaging*.

[44] Sardari A, Tabarsi P, Borhany H, Mohiaddin R, Houshmand G (2021). Myocarditis detected after COVID-19 recovery. *European Heart Journal - Cardiovascular Imaging*.

[45] Kim HW, Jenista ER, Wendell DC, Azevedo CF, Campbell MJ, Darty SN (2021). Patients with Acute Myocarditis Following mRNA COVID-19 Vaccination. *JAMA Cardiology*.

[46] Bozkurt B, Kamat I, Hotez PJ (2021). Myocarditis with COVID-19 mRNA Vaccines. *Circulation*.

[47] Montgomery J, Ryan M, Engler R, Hoffman D, McClenathan B, Collins L (2021). Myocarditis Following Immunization with mRNA COVID-19 Vaccines in Members of the us Military. *JAMA Cardiology*.

[48] Simone A, Herald J, Chen A, Gulati N, Shen AY, Lewin B (2021). Acute Myocarditis Following COVID-19 mRNA Vaccination in Adults Aged 18 Years or Older. *JAMA Internal Medicine*.

[49] Chen T, Wu D, Chen H, Yan W, Yang D, Chen G (2020). Clinical characteristics of 113 deceased patients with coronavirus disease 2019: retrospective study. *British Medical Journal*.

[50] Nejadrahim R, Khademolhosseini S, Kavandi H, Hajizadeh R (2021). Severe acute respiratory syndrome coronavirus-2- or pregnancy-related cardiomyopathy, a differential to be considered in the current pandemic: a case report. *Journal of Medical Case Reports*.

[51] Garg S, Singh A, Kalita M, Siddiqui AZ, Kapoor MC (2020). Peripartum cardiomyopathy mimicking COVID-19 infection. *Journal of Anaesthesiology Clinical Pharmacology*.

[52] Bhattacharyya PJ, Attri PK, Farooqui W (2020). Takotsubo cardiomyopathy in early term pregnancy: a rare cardiac complication of SARS-CoV-2 infection. *BMJ Case Reports*.

[53] Murthy H, Iqbal M, Chavez JC, Kharfan-Dabaja MA (2019). Cytokine Release Syndrome: Current Perspectives. *ImmunoTargets and Therapy*.

[54] Nef HM, Möllmann H, Akashi YJ, Hamm CW (2010). Mechanisms of stress (Takotsubo) cardiomyopathy. *Nature Reviews Cardiology*.

[55] Sachdeva J, Dai W, Kloner RA (2014). Functional and Histological Assessment of an Experimental Model of Takotsubo’s Cardiomyopathy. *Journal of the American Heart Association*.

[56] Ye Q, Wang B, Mao J (2020). The pathogenesis and treatment of the ‘Cytokine Storm’ in COVID-19. *Journal of Infection*.

[57] McGonagle D, Sharif K, O’Regan A, Bridgewood C (2020). The Role of Cytokines including Interleukin-6 in COVID-19 induced Pneumonia and Macrophage Activation Syndrome-Like Disease. *Autoimmunity Reviews*.

[58] De Vita S, Ippolito S, Caracciolo MM, Barosi A (2020). Peripartum cardiomyopathy in a COVID‐19‐infected woman: differential diagnosis with acute myocarditis—a case report from a Hub Institution during the COVID‐19 outbreak. *Echocardiography*.

[59] Giustino G, Croft LB, Oates CP, Rahman K, Lerakis S, Reddy VY (2020). Takotsubo Cardiomyopathy in COVID-19. *Journal of the American College of Cardiology*.

[60] Minhas AS, Scheel P, Garibaldi B, Liu G, Horton M, Jennings M (2020). Takotsubo Syndrome in the Setting of COVID-19. *JACC: Case Reports*.

[61] Sultan AA, West J, Tata LJ, Fleming KM, Nelson-Piercy C, Grainge MJ (2012). Risk of first venous thromboembolism in and around pregnancy: a population-based cohort study. *British Journal of Haematology*.

[62] Abdul Sultan A, West J, Tata LJ, Fleming KM, Nelson-Piercy C, Grainge MJ (2013). Risk of first venous thromboembolism in pregnant women in hospital: population based cohort study from England. *British Medical Journal*.

[63] Klok FA, Kruip MJHA, van der Meer NJM, Arbous MS, Gommers DAMPJ, Kant KM (2020). Incidence of thrombotic complications in critically ill ICU patients with COVID-19. *Thrombosis Research*.

[64] Cui S, Chen S, Li X, Liu S, Wang F (2020). Prevalence of venous thromboembolism in patients with severe novel coronavirus pneumonia. *Journal of Thrombosis and Haemostasis*.

[65] Malas MB, Naazie IN, Elsayed N, Mathlouthi A, Marmor R, Clary B (2020). Thromboembolism risk of COVID-19 is high and associated with a higher risk of mortality: a systematic review and meta-analysis. *EClinicalMedicine*.

[66] Martinelli I, Ferrazzi E, Ciavarella A, Erra R, Iurlaro E, Ossola M (2020). Pulmonary embolism in a young pregnant woman with COVID-19. *Thrombosis Research*.

[67] Mohammadi S, Abouzaripour M, Hesam Shariati N, Hesam Shariati MB (2020). Ovarian vein thrombosis after coronavirus disease (COVID-19) infection in a pregnant woman: case report. *Journal of Thrombosis and Thrombolysis*.

[68] Houghton DE, Wysokinski W, Casanegra AI, Padrnos LJ, Shah S, Wysokinska E (2022). Risk of venous thromboembolism after COVID‐19 vaccination. *Journal of Thrombosis and Haemostasis*.

[69] Kadir RA, Kobayashi T, Iba T, Erez O, Thachil J, Kazi S (2020). COVID‐19 coagulopathy in pregnancy: Critical review, preliminary recommendations, and ISTH registry—Communication from the ISTH SSC for Women’s Health. *Journal of Thrombosis and Haemostasis*.

[70] Devis P, Knuttinen MG (2017). Deep venous thrombosis in pregnancy: incidence, pathogenesis and endovascular management. *Cardiovascular Diagnosis and Therapy*.

[71] Gutiérrez García I, Pérez Cañadas P, Martínez Uriarte J, García Izquierdo O, Angeles Jódar Pérez M, García de Guadiana Romualdo L (2018). D-dimer during pregnancy: establishing trimester-specific reference intervals. *Scandinavian Journal of Clinical and Laboratory Investigation*.

[72] (2020). Gestational Hypertension and Preeclampsia: ACOG Practice Bulletin Summary, Number 222. *Obstetrics & Gynecology*.

[73] Dashraath P, Wong JLJ, Lim MXK, Lim LM, Li S, Biswas A (2020). Coronavirus disease 2019 (COVID-19) pandemic and pregnancy. *American Journal of Obstetrics and Gynecology*.

[74] Di Mascio D, Khalil A, Saccone G, Rizzo G, Buca D, Liberati M (2020). Outcome of coronavirus spectrum infections (SARS, MERS, COVID-19) during pregnancy: a systematic review and meta-analysis. *American Journal of Obstetrics & Gynecology MFM*.

[75] Ahmed A, Rezai H, Broadway-Stringer S (2017). Evidence-Based Revised View of the Pathophysiology of Preeclampsia. *Advances in Experimental Medicine and Biology*.

[76] Mendoza M, Garcia‐Ruiz I, Maiz N, Rodo C, Garcia‐Manau P, Serrano B (2020). Pre‐eclampsia‐like syndrome induced by severe COVID‐19: a prospective observational study. *BJOG: An International Journal of Obstetrics & Gynaecology*.

[77] Wei SQ, Bilodeau-Bertrand M, Liu S, Auger N (2021). The impact of COVID-19 on pregnancy outcomes: a systematic review and meta-analysis. *Canadian Medical Association Journal*.

[78] Ahmed I, Eltaweel N, Antoun L, Rehal A (2020). Severe pre-eclampsia complicated by acute fatty liver disease of pregnancy, HELLP syndrome and acute kidney injury following SARS-CoV-2 infection. *BMJ Case Reports*.

[79] Hansen JN, Hine J, Strout TD (2021). COVID-19 and preeclampsia with severe features at 34-weeks gestation. *The American Journal of Emergency Medicine*.

[80] Sinkey RG, Rajapreyar I, Robbins LS, Dionne-Odom J, Pogwizd SM, Casey BM (2020). Heart Failure with Preserved Ejection Fraction in a Postpartum Patient with Superimposed Preeclampsia and COVID-19. *American Journal of Perinatology Reports*.

[81] Papageorghiou AT, Deruelle P, Gunier RB, Rauch S, García-May PK, Mhatre M (2021). Preeclampsia and COVID-19: results from the INTERCOVID prospective longitudinal study. *American Journal of Obstetrics and Gynecology*.

[82] Negro A, Fama A, Penna D, Belloni L, Zerbini A, Giuri PG (2021). SFLT‐1 levels in COVID‐19 patients: Association with outcome and thrombosis. *American Journal of Hematology*.

[83] Davidson KW, Barry MJ, Mangione CM, Cabana M, Caughey AB, Davis EM (2021). Aspirin Use to Prevent Preeclampsia and Related Morbidity and Mortality US Preventive Services Task Force Recommendation Statement. *Journal of the American Medical Association*.

[84] Cheng P, Zhu H, Witteles RM, Wu JC, Quertermous T, Wu SM (2020). Cardiovascular Risks in Patients with COVID-19: Potential Mechanisms and Areas of Uncertainty. *Current Cardiology Reports*.

[85] Bhatla A, Mayer MM, Adusumalli S, Hyman MC, Oh E, Tierney A (2020). COVID-19 and cardiac arrhythmias. *Heart Rhythm*.

[86] Babapoor-Farrokhran S, Rasekhi RT, Gill D, Babapoor S, Amanullah A (2020). Arrhythmia in COVID-19. *SN Comprehensive Clinical Medicine*.

[87] Center for Disease Control and Prevention (CDC) Morbidity and Mortality Weekly Report (MMWR): Trends in disease severity and health care utilization during the early omicron variant period compared with previous SARS-CoV-2 High Transmission Periods – United States, December 2020-January 2022. https://www.cdc.gov/mmwr/volumes/71/wr/mm7104e4.htm.

[88] (2020). March of Dimes. COVID-19 Maternal and Infant Health Research Registries. https://www.marchofdimes.org/research/covid19-maternal-and-infant-health-research-registries.aspx.

[89] University of California San Francisco (2021). Priority: pregnancy coronavirus otucomes registry: PRIORITY STUDY. https://priority.ucsf.edu.

[90] (2021). INTERCOVID. https://coronavirus.tghn.org/covid-disease-characterisation/covid-19-pregnancy/.

